# Integrated bioinformatics analysis and experimental validation of *SRF* as a potential senescence biomarker in MASLD

**DOI:** 10.3389/fmolb.2026.1745039

**Published:** 2026-04-29

**Authors:** Mengyue Li, Chang Gao, Yuhui Shi, Wenyu Peng, Yuan Yin, Bin Lu, Zhang Fu

**Affiliations:** 1 Department of Infectious Disease, The Seventh Affiliated Hospital of Sun Yat-Sen University, Shenzhen, China; 2 Tomas Lindahl Nobel Laureate Laboratory, The Seventh Affiliated Hospital of Sun Yat-sen University, Shenzhen, China; 3 Department of Hepatobiliary Surgery, The Third Affiliated Hospital of Sun Yat-Sen University, Guangzhou, China; 4 Gastric Cancer Center, West China Hospital, and State Key Laboratory of Biotherapy, Sichuan University, Chengdu, Sichuan, China; 5 Department of Geriatrics, The Seventh Affiliated Hospital of Sun Yat-Sen University, Shenzhen, China

**Keywords:** cellular senescence, machine learning, MASLD, SRF, WGCNA

## Abstract

**Background:**

Metabolic dysfunction-associated steatotic liver disease (MASLD) is closely linked to cellular senescence. Identifying senescence-related biomarkers is crucial for discovering potential diagnostic markers and therapeutic strategies for MASLD.

**Methods:**

Senescence-related genes from GenAge were integrated with GEO datasets (GSE89632, GSE63067). Overlapping genes among differentially expressed genes, WGCNA modules, and senescence sets were screened using five machine learning algorithms (LASSO, Random Forest, Boruta, GBM, SVM-RFE). Immune infiltration and functional enrichment were assessed by single-sample gene set enrichment analysis (ssGSEA) and gene set enrichment analysis (GSEA), respectively. Finally, the expression of these four hub genes was confirmed by RT-qPCR in animal models, cell lines, and clinical samples, while SRF protein levels were further validated by Western blotting. Single-cell RNA sequencing (scRNA-seq) was utilized for cell-type-specific characterization and *in silico* virtual knockout of *SRF*. The pathogenic role of *SRF* was investigated through *in vitro* knockdown assays.

**Results:**

A total of 46 genes associated with both MASLD and cellular senescence were identified. After screening by ML and validation with external datasets, *SRF, ATF3, ME1,* and *GADD45G* were determined to be key genes. Experimental validation demonstrated significantly downregulated expression of *SRF, ATF3, and GADD45G* in MASLD, whereas *ME1* was upregulated. Single-cell analysis confirmed predominant expression of *SRF* in hepatocytes, with marked downregulation during MASLD progression. Furthermore, *in silico* virtual knockout of *SRF* in single-cell clusters revealed a dramatic transcriptomic shift toward a pro-steatotic and pro-senescence state, consistent with the observed bulk RNA-seq profiles. Critically, *SRF* knockdown significantly exacerbated both lipid deposition and cellular senescence in hepatocyte steatosis models, confirming its functional involvement in MASLD pathogenesis.

**Conclusion:**

*SRF* deficiency accelerates hepatocellular senescence and exacerbates MASLD progression. Our results identify *SRF* as a potential biomarker and therapeutic target for MASLD.

## 1 Introduction

Metabolic dysfunction–associated steatotic liver disease (MASLD), formerly known as non-alcoholic fatty liver disease (NAFLD), affects approximately one-third of adults worldwide and represents the fastest-growing cause of chronic liver disease and hepatocellular carcinoma (HCC) globally (Danpanichkul et al., 2025; Rinella et al., 2023). Recent epidemiological data indicate that about 38% of the global adult population is affected by MASLD, and this prevalence is projected to increase to 55% by 2040 (Younossi et al., 2025). MASLD represents the hepatic manifestation of metabolic syndrome, characterized by excessive fat accumulation (≥5%) in hepatocytes without excessive alcohol consumption (Liu et al., 2024). MASLD can progress to metabolic dysfunction–associated steatohepatitis (MASH), which is characterized by liver inflammation and renders affected individuals susceptible to fibrosis, cirrhosis, HCC, and liver failure (Newsome and Loomba, 2025). Although early treatment may facilitate complete recovery, approximately 20% of MASLD patients progress to cirrhosis and end-stage liver disease, severely endangering human health (Chalasani et al., 2018). Despite being a global health issue, therapeutic options for MASLD and its related diseases remain highly limited (Zhang et al., 2025). Moreover, MASLD is not only a major cause of liver-related morbidity and mortality but also influences the progression of other chronic metabolic diseases, including type 2 diabetes mellitus (T2DM), atherosclerotic cardiovascular diseases, and extrahepatic malignancies (Cai et al., 2025). Therefore, identifying robust biomarkers for the early screening and risk stratification of MASLD patients is an urgent priority.

Cellular senescence (CS) is a stable programmed response characterized by irreversible cell cycle arrest and the secretion of a senescence-associated secretory phenotype (SASP) (Sanfeliu-Redondo et al., 2024; Ge et al., 2023). In the context of MASLD, which represents a continuous spectrum from simple steatosis (NAFL) to MASH, the role of CS is complex and exhibits context-dependent heterogeneity. CS functions as a “double-edged sword”: on one hand, it acts as a secondary protective mechanism in activated hepatic stellate cells (HSCs), limiting fibrosis (Krizhanovsky et al., 2008); on the other hand, it serves as a primary driver in hepatocytes (Ogrodnik et al., 2017). In hepatocytes, CS impairs mitochondrial β-oxidation and generates reactive oxygen species (ROS), thereby blocking fatty acid elimination and promoting triglyceride accumulation (Matsuzawa-Nagata et al., 2008; Meijnikman et al., 2021). Consequently, the accumulation of senescent cells and the SASP-mediated inflammatory microenvironment drive the transition from simple steatosis to MASH and fibrosis (Gong et al., 2025).

Despite these insights, the specific regulatory mechanisms of CS across the MASLD spectrum remain incompletely understood, and there is a lack of robust biomarkers that function independently of this heterogeneity. Therefore, screening for core cellular senescence biomarkers associated with MASLD is crucial. It will not only deepen our understanding of MASLD pathogenesis but also provide potential targets for early diagnosis and therapy. Against this backdrop, this study aims to identify key CS-related biomarkers in MASLD through the integration of bioinformatics analysis and experimental validation, providing new insights into the molecular mechanisms and suggesting candidate targets for future diagnostic and therapeutic strategies.

In this study, based on differential expression analysis and weighted gene coexpression network analysis (WGCNA), we identified 46 genes associated with both MASLD and CS. To ensure robust biomarker selection, we employed an integrated machine learning (ML) framework combining five distinct algorithms—least absolute shrinkage and selection operator (LASSO), random forest (RF), Boruta, Gradient Boosting Machine (GBM), and Support Vector Machine-Recursive Feature Elimination (SVM-RFE)—to screen for core signatures. The results demonstrated that *SRF*, *ATF3*, *ME1*, and *GADD45G* are closely associated with cellular senescence in MASLD. Finally, the expression of these hub genes was validated in animal models, cell lines, and clinical samples, and the critical functional role of the core target *SRF* was further elucidated through *in vitro* knockdown assays.

## 2 Materials and methods

### 2.1 Data collection, preprocessing and differentially expressed genes (DEGs) screening

**FIGURE 1:**
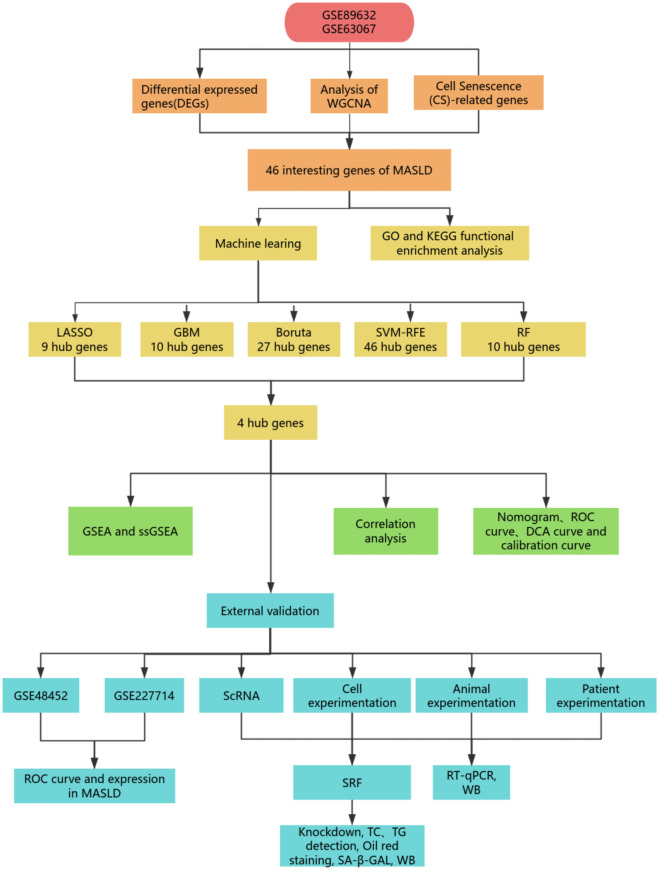
Flowchart of the study.

Gene expression profile data were obtained from the Gene Expression Omnibus (GEO; http://www.ncbi.nlm.nih.gov/geo) database. A total of 4 MASLD-related datasets were included in this study: GSE89632, GSE63067, GSE48452, and GSE227714. GSE89632 and GSE63067 were both derived from human samples and were merged to serve as the training set; the validation set included the human-derived GSE48452 dataset and the rat-derived GSE227714 dataset. Detailed sample information for each dataset is shown in Table 1. In this study, after the GSE63067 and GSE89632 datasets were merged, the Combat algorithm in the SVA package was used for batch effect correction, and then the R package “Limma” was applied for differential expression analysis. The overall research workflow is illustrated in Figure 1. Differential expression analysis was performed with the Limma package, with an adjusted *P-*value (adj.*P*) < 0.05 and |log_2_ (fold change)| ≥ 0.585. Volcano plots and heatmaps were generated to visualize DEG distribution and expression patterns.

**TABLE 1:** Fundamental details of the GEO datasets utilized in this research.

GEO series	Platform	Organism	Samples		Category
			Normal	MASLD	
GSE89632	GPL14951	*Homo sapiens*	24	39	Training set
GSE63067	GPL570	*Homo sapiens*	7	11	Training set
GSE48452	GPL11532	*Homo sapiens*	14	18	Validation set
GSE227714	GPL22740	*Rattus norvegicus*	10	10	Validation set

### 2.2 WGCNA and identification of cellular senescence-associated DEGs (CSDEGs)

A gene co-expression network was constructed *via* the WGCNA package. After excluding outlier samples, the optimal soft threshold (*β* = 6) was determined based on scale independence and average connectivity. Genes were clustered into modules using the “dynamic tree cutting” algorithm, and the brown (953 genes) and blue (1,210 genes) modules, which showed the highest correlation with MASLD, were selected as core modules. A total of 866 senescence-related genes were downloaded from the GenAge database (Supplementary Table S1). The intersection of DEGs, core module genes, and cell senescence-related genes was analyzed to identify 46 CS-DEGs.

### 2.3 Functional enrichment analysis and ML-based hub gene screening

Functional enrichment analysis (GO and KEGG) of the 46 CS-DEGs was conducted using the clusterProfiler package (*P* < 0.05). To identify the most robust hub genes, we employed an ensemble machine-learning framework comprising five distinct algorithms, each with specific strategies to mitigate overfitting. Specifically, LASSO regression utilized 10-fold cross-validation to select the optimal penalty parameter (λ). SVM-RFE employed 10-fold cross-validation for recursive feature elimination to enhance the stability of feature ranking. For Random Forest (RF) and GBM, model complexity was regulated based on Out-of-Bag (OOB) error and deviance to prevent over-training. Additionally, the Boruta algorithm was used to filter out stochastic noise by comparing the importance of real features against shadow features through multiple iterations. The intersection of genes identified by all five algorithms yielded 4 core hub genes: *SRF*, *ATF3*, *ME1*, and *GADD45G*.

### 2.4 Immune infiltration analysis and gene set enrichment analysis (GSEA)

The single-sample GSEA (ssGSEA) algorithm was used to calculate the relative abundance of 24 immune cell types in the training set, and correlation analysis was performed to explore associations between hub genes and immune cell infiltration. GSEA was also conducted to identify enriched KEGG pathways and GO terms for the hub genes to clarify their potential mechanisms in MASLD.

### 2.5 Evaluation of the diagnostic value

The diagnostic efficacy of the 4 hub genes for MASLD was evaluated using ROC curves (pROC package) and AUC values. External validation was performed using the GSE48452 and GSE227714 datasets. A MASLD risk prediction nomogram was constructed based on the hub genes using the rms package. Calibration curves and decision curve analysis (DCA) were used to assess the nomogram’s prediction accuracy and clinical application value.

### 2.6 Single-cell RNA sequencing analysis

In this study, we performed *de novo* scRNA-seq analysis on liver specimens from three control mice and 3 MASLD model mice. The scRNA-seq data processing workflow was as follows: First, high-quality cells were filtered with mitochondrial gene proportion <20% and gene expression counts between 500 and 4,000. Subsequently, an integrated workflow was implemented *via* the Seurat pipeline. The remaining cells were further scaled and normalized using the “LogNormalize” method combined with a linear regression model, and the top 2000 highly variable genes were identified through the “FindVariableFeatures” function. Dimensionality reduction of the scRNA-seq data was then performed by principal component analysis (PCA). Uniform Manifold Approximation and Projection (UMAP) dimensionality reduction, dataset integration, and cell type annotation were conducted with the assistance of the R package “SingleR”. To eliminate batch effects between samples, soft k-means clustering was performed using the “Harmony” software. Cell clustering was carried out using the “FindClusters” function with the resolution parameter set to 1. The annotation of cell clusters was based on genes with elevated expression levels, genes exhibiting unique expression patterns, and well-documented canonical cell markers.

To predict the regulatory influence of *Srf* on the hepatic transcriptomic landscape, we performed *in silico* virtual knockout (vKO) analysis using the scTenifoldKnk (v1.0.1) pipeline. Briefly, a multi-layered gene regulatory network (PCNet) was constructed by subsampling cells from the MASLD group. The regulatory weight of Srf was then set to zero to simulate a loss-of-function mutation. The resulting transcriptomic perturbations were quantified using manifold alignment, with downstream functional implications assessed *via* KEGG pathway enrichment analysis.

### 2.7 Establishment of the MASLD animal model and sample

Male C57BL/6J mice (6–8 weeks old) were procured from the Animal Center of Sun Yat-sen University and randomly divided into a normal diet (NCD) group and a high-fat diet (HFD) group (*n* = 5 per group). After 8 weeks of feeding, mice were euthanized, all animals were humanely euthanized via intraperitoneal administration of pentobarbital sodium at a dosage of 200 mg/kg. The livers were excised, rinsed with normal saline, blotted dry, and fixed in 4% paraformaldehyde for H&E staining or stored at −80 °C for molecular detection. The animal protocol was approved by the Animal Ethics Committee of the third affiliated hospital of Sun Yat-sen University (No. SYSU-IACUC-2025-001543).

### 2.8 Cell model and *SRF* knockdown

HepG2 cell line used in this investigation was supplied by the Liver Disease Center Laboratory of the Third Affiliated Hospital of Sun Yat-sen University. HepG2 cells were cultured in high-glucose DMEM (with 10% FBS, 100 U/mL penicillin, and 100 μg/mL streptomycin) at 37 °C with 5% CO_2_. The MASLD cell model was established by treating cells with 0.2 mM palmitic acid (PA) + 0.4 mM oleic acid (OA) for 24 h. *SRF* knockdown was achieved *via* transfection with human *SRF* siRNA (Sangon Biotech, China) using Lipo 2000 (GLPBIO, Montclair, United States, rj Lipo 2000). TC and TG levels in cells were measured with commercial kits (#A110, #A111, Jiancheng Bioengineering Institute, Nanjing, China). The siRNA sequences are provided in Table 2.

**TABLE 2:** The list of siRNA sequences.

Genes	Species	Target sequence (5'- 3′)
*SRF* siRNA sense	Human	GCAAGGCACUGAUUCAGACTT
*SRF* siRNA antisense	Human	GUCUGAAUCAGUGCCUUGCT

### 2.9 Detection of gene expression by RT‒qPCR

Liver tissue samples from 6 healthy controls and 11 MASLD patients (approved by the Ethics Committee of West China Hospital, No. 137 (2020)) were collected. Total RNA was extracted from clinical samples, mouse liver tissues, and HepG2 cells. RT-qPCR was used to detect the relative expression of hub genes (TIANLONG: Gentier 96E). The sequences of primers used for RT‒qPCR are shown in Table 3. Western blotting was performed to measure SRF protein levels.

**TABLE 3:** Gene-specific sequences of primers used for RT‒qPCR.

Primer	Sequence (5’ - 3′)
Hu-*SRF*-F	TCACCTACCAGGTGTCGGAGTC
Hu-*SRF*-R	GTGCTGTTTGGATGGTGGAGGT
Hu-*ATF3*-F	CGCTGGAATCAGTCACTGTCAG
Hu-*ATF3*-R	CTTGTTTCGGCACTTTGCAGCTG
Hu-*ME1*-F	GGAGTTGCTCTTGGTGTTGTGG
Hu-*ME1*-R	GGATAAAGCCGACCCTCTTCCA
Hu-*GADD45G*-F	CGTCTACGAGTCAGCCAAAGTC
Hu-*GADD45G*-R	CGATGTCGTTCTCGCAGCAGAA
Hu-*TP53*-F	CCTCAGCATCTTATCCGAGTGG
Hu-*TP53*-R	TGGATGGTGGTACAGTCAGAGC
Hu-*CDKN1A*-F	AGGTGGACCTGGAGACTCTCAG
Hu-*CDKN1A*-R	TCCTCTTGGAGAAGATCAGCCG
Hu-*CDKN2A*-F	CTCGTGCTGATGCTACTGAGGA
Hu-*CDKN2A*-R	GGTCGGCGCAGTTGGGCTCC
Hu-*β-actin*-F	CACCATTGGCAATGAGCGGTTC
Hu-*β-actin*-R	AGGTCTTTGCGGATGTCCACGT
M-*Srf*-F	CTCACCTACCAGGTGTCGGAAT
M-*Srf*-R	CTGCTGACTTGCATGGTGGTAG
M-*Atf3*-F	GAAGATGAGAGGAAAAGGAGGCG
M-*Atf3*-R	GCTCAGCATTCACACTCTCCAG
M-*Me1*-F	AGAGCAGTGCTACAAGGTGACC
M-*Me1*-R	CCAAGAGCAACTCCAGGGAACA
M-*Gadd45g*-F	TCTACGAGTCCGCCAAAGTCCT
M-*Gadd45g*-R	CTCACAGCAGAACGCCTGAATC
M-*β-actin*-F	CATTGCTGACAGGATGCAGAAGG
M-*β-actin*-R	TGCTGGAAGGTGGACAGTGAGG

### 2.10 Statistical analysis

Data were analyzed and visualized using GraphPad Prism 9.5. Measurement data are presented as mean ± SEM. The unpaired Student’s t-test was used for comparisons between two groups (normal distribution, homogeneous variance), and ANOVA followed by the Student–Newman–Keuls method was used for multiple group comparisons. Statistical significance was set at *P* < 0.05. All experiments were independently repeated 3 times.

## 3 Results

### 3.1 Identification of DEGs

**FIGURE 2:**
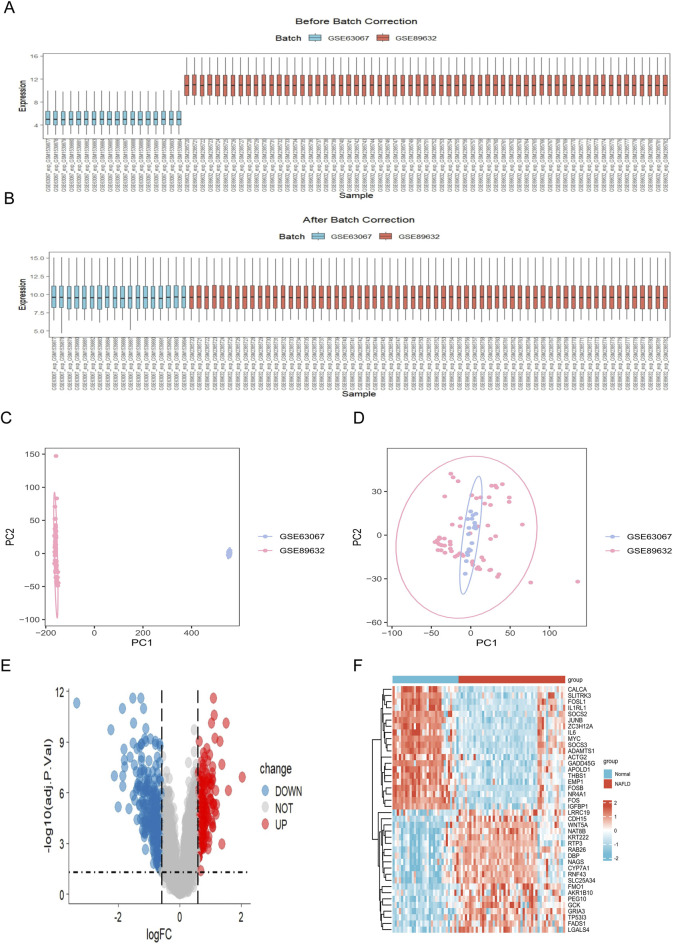
Elimination of batch effects and identification of DEGs in MASLD. **(A,B)** Box plots visualizing the distribution of gene expression profiles for the GSE63067 (blue) and GSE89632 (red) datasets **(A)** before and **(B)** after batch effect correction. The alignment of medians in **(B)** indicates successful normalization. **(C,D)** PCA plots assessing the sample distribution. **(C)** Before correction, samples clustered distinctly by dataset source, indicating a significant batch effect. **(D)** After application of the ComBat algorithm, samples from both datasets were well-integrated, confirming the removal of batch effects. **(E)** Volcano plot displaying the 589 DEGs between MASLD and control samples. Red dots represent 231 upregulated genes, blue dots represent 358 downregulated genes, and grey dots indicate no significant difference (adjusted *P* value <0.05 and |log_2_FC| ≥ 0.585). **(F)** Hierarchical clustering heatmap of the top 20 upregulated and top 20 downregulated DEGs. The dendrogram on the left illustrates the hierarchical clustering of genes. DEGs, differentially expressed genes; FC, fold change; MASLD, metabolic dysfunction-associated steatotic liver disease; PCA, principal component analysis.

In this study, two datasets (GSE89632 and GSE63067) were integrated, encompassing a total of 31 normal samples and 50 MASLD case samples. First, the ‘cbind ()` function in R was used to merge the datasets, and the sva package was applied to correct for batch effects. Box plots demonstrated the normalization of gene expression distributions before and after correction (Figures 2A,B). Furthermore, principal component analysis (PCA) was performed to assess sample integration; samples clustered distinctly by dataset origin before correction (Figure 2C), whereas the corrected data exhibited a well-integrated distribution, with significant batch effects effectively eliminated (Figure 2D), confirming the validity of the merged dataset for downstream analysis. Subsequently, differential expression analysis was performed *via* the Limma package, leading to the identification of 589 DEGs between MASLD patients and controls (Supplementary Table S2). Among these DEGs, 231 genes were upregulated and 358 genes were downregulated in the MASLD group. Figure 2E presents the overall distribution of DEGs in the form of a volcano plot, while Figure 2F displays the top 20 upregulated and downregulated DEGs with the most significant expression changes *via* a heatmap, intuitively reflecting the expression patterns of key genes closely associated with the development and progression of MASLD.

### 3.2 WGCNA and identification of key modules

**FIGURE 3:**
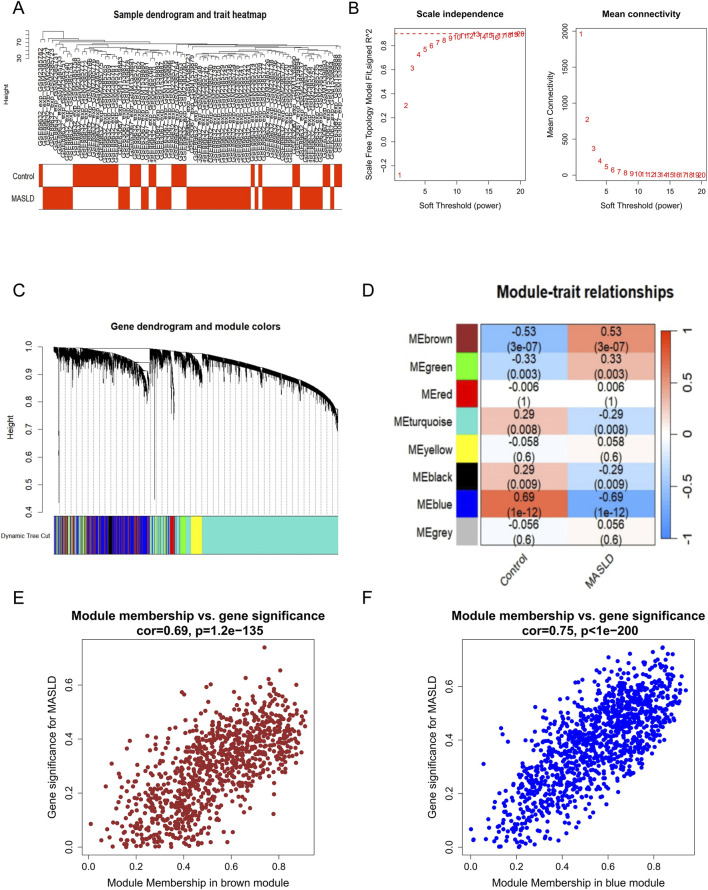
Construction of the WGCNA and identification of key modules associated with MASLD. **(A)** Sample clustering dendrogram with a trait heatmap underneath. The color bands represent the clinical traits (Normal vs. MASLD) associated with each sample cluster, ensuring no significant outliers were present. **(B)** Analysis of the network topology for various soft-thresholding powers. The left panel shows the scale-free fit index (*R* ^*2*^) *versus* soft-thresholding power (*β*), and the right panel displays the mean connectivity. A power of *β* = 6 was selected to ensure a scale-free network distribution (scale-free *R* ^*2*^ = 0.9). **(C)** Cluster dendrogram of differentially expressed genes. The color row underneath the tree indicates the module assignment identified by the dynamic tree cut method. **(D)** Module-trait relationship heatmap. Each row corresponds to a color-coded module, and columns represent clinical traits. The numbers in each cell indicate the correlation coefficient and the corresponding *P*-value. The blue (*r* = −0.69, *P* = 1e-12) and brown (*r* = 0.53, *P* = 3e-7) modules show the highest significant correlations with MASLD. **(E)** Scatter plot of MM *versus* GS for the brown module (cor = 0.69, *P* < 1.2e-135), indicating a strong association with the disease phenotype. **(F)** Scatter plot of MM *versus* GS for the blue module (cor = 0.75, *P* < 1e-200), identifying it as another key module for MASLD. GS, gene significance; MM, module membership; MASLD, metabolic dysfunction-associated steatotic liver disease; WGCNA, weighted gene coexpression network analysis.

We constructed a scale-free coexpression network *via* WGCNA with the aim of identifying modules with the highest correlation. First, a sample clustering dendrogram was generated to detect outliers and ensure sample quality (Figure 3A). Based on the analysis of scale independence and mean connectivity, the optimal soft-thresholding power (*β* = 6, scale-free *R* ^2^ = 0.9) was subsequently selected (Figure 3B). Subsequently, a total of 8 distinct gene modules were identified using the dynamic tree cutting method (Figure 3C). To explore the relationship between these modules and MASLD, we performed a module-trait correlation analysis. The heatmap results demonstrated that the brown module (*r* = 0.53, *P* < 0.001) and the blue module (*r* = −0.69, *P* < 0.001) exhibited the highest correlations with the MASLD phenotype (Figure 3D). Therefore, the brown module (containing 953 genes) and the blue module (containing 1,210 genes) were selected for subsequent analyses. In addition, correlation analysis between module membership (MM) and gene significance (GS) revealed that both modules presented a significant positive correlation: cor = 0.69 (*P* < 0.001) for the brown module (Figure 3E) and cor = 0.75 (*P* < 0.001) for the blue module (Figure 3F).

### 3.3 Functional enrichment analysis

**FIGURE 4:**
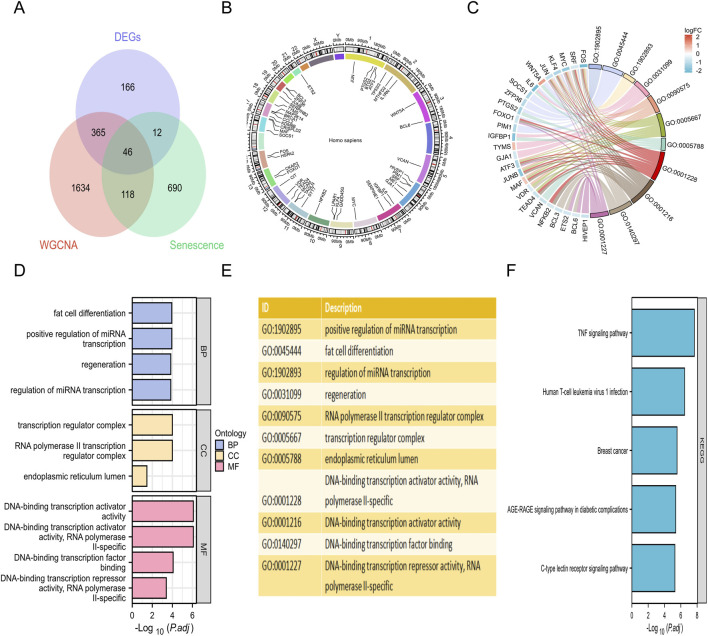
Identification and functional enrichment analysis of senescence-related genes in MASLD. **(A)** Venn diagram showing intersection of DEGs (*n* = 589), WGCNAGs (*n* = 2,163), and CSRGs (*n* = 866), identifying 46 candidate genes (CGs). **(B)** Circos plot visualizing the chromosomal distribution of the 46 overlapping genes. The outer ring represents human chromosomes, and gene labels indicate their specific genomic locations. **(C)** Chord diagram displaying the detailed relationship between the candidate genes (left semicircle) and their corresponding enriched GO terms (right semicircle), highlighting the involvement of genes in multiple biological functions. **(D)** Bar chart showing the top enriched GO terms categorized by BP, CC, and MF. The x-axis represents the significance level, calculated as -log_10_ (adjusted *P* value). **(E)** A descriptive list of the GO term IDs and functional descriptions corresponding to the enrichment results shown in panels **(C,D)**, **(F)** Bar chart showing the top enriched KEGG pathways. The length of the bars corresponds to statistical significance (-log_10_ (adjusted *P* value)). BP, biological process; CC, cellular component; CSRGs, cell senescence related genes; DEGs, differentially expressed genes; GO, gene ontology; KEGG, kyoto encyclopedia of genes and genomes; MF, molecular function; MASLD, metabolic dysfunction-associated steatotic liver disease; WGCNAGs, WGCNA-derived gene.

To identify robust senescence-associated biomarkers in MASLD, we performed an intersection analysis of the identified DEGs, the key WGCNA module genes, and the cellular senescence-related genes (CSRGs). This analysis yielded 46 overlapping candidate genes (Figure 4A). The chromosomal distribution and specific genomic loci of these genes were visualized *via* a Circos plot (Figure 4B). To further elucidate the potential biological functions and regulatory mechanisms of these overlapping genes in MASLD progression, we conducted GO and KEGG enrichment analyses with a significance threshold of adjusted *P*-value <0.05. The GO analysis results revealed distinct functional enrichments across three categories (Figures 4C–E). In terms of BP, the genes were primarily enriched in ‘fat cell differentiation’, ‘positive regulation of miRNA transcription’, and ‘regeneration’. Regarding CC, significant enrichment was observed in the ‘transcription regulator complex’, ‘RNA polymerase II transcription regulatory complex’, and ‘endoplasmic reticulum lumen’. For MF, the genes were predominantly associated with ‘DNA-binding transcription activator activity’. Furthermore, KEGG pathway analysis highlighted the involvement of these genes in critical inflammatory and metabolic pathways, specifically the ‘TNF signaling pathway’ and the ‘AGE-RAGE signaling pathway in diabetic complications’ (Figure 4F).

### 3.4 Identification of candidate diagnostic genes via ML

**FIGURE 5:**
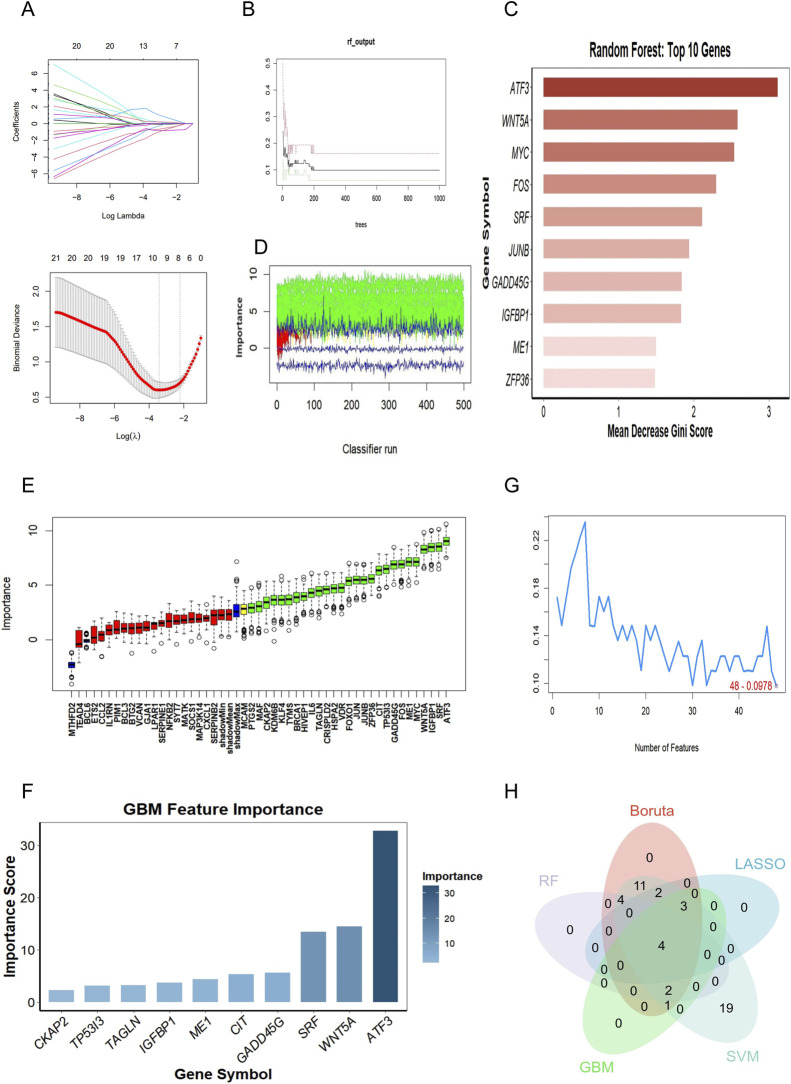
Screening and identification of robust diagnostic biomarkers using five machine learning algorithms. **(A)** LASSO regression analysis. The upper panel displays the coefficients of candidate genes with varying penalty parameters (λ). The lower panel shows the selection of the optimal λ *via* 10-fold cross-validation. The vertical dotted line marks the λ_min_ value, which was determined to be 0.0392 (logλ = −3.239), identifying the optimal gene subset. **(B)** RF model construction. The plot illustrates the relationship between the number of decision trees (x-axis) and the out-of-bag error rate (y-axis). The error rate stabilizes as the number of trees increases, with 500 trees selected as the optimal parameter. **(C)** RF feature importance. The top candidate genes ranked by the MeanDecreaseGini index. The top 10 features (all with MeanDecreaseGini scores >1.3) were selected as candidate diagnostic markers. **(D)** Dynamic profile of Boruta feature selection. The plot tracks the importance of attributes (genes) over iterations. Green lines represent confirmed important features, red lines represent rejected features, and blue lines represent shadow features (randomized controls). **(E)** Final importance of Boruta-confirmed features. Box plots showing the final importance of the confirmed features identified by the Boruta algorithm. **(F)** GBM analysis. The bar chart displays the relative importance of the top 10 genes identified by the GBM algorithm. **(G)** SVM-RFE analysis. The curve represents the accuracy/error rate of the SVM-RFE algorithm with varying numbers of features. The red text indicates the point of optimal feature selection (lowest error or highest accuracy). **(H)** Venn diagram of diagnostic markers. The intersection of potential biomarkers identified by the five algorithms. The central overlap confirms SRF, ATF3, ME1, and GADD45G as the four robust hub genes shared across all models. GBM, Gradient Boosting Machine; LASSO, least absolute shrinkage and selection operator; RF, random forest; SVM-RFE, support vector machine-recursive feature elimination.

To identify robust diagnostic biomarkers with high reliability, we adopted an integrated ML approach combining five distinct algorithms: LASSO, RF, Boruta, GBM, and SVM-RFE. First, LASSO regression was performed with 10-fold cross-validation, and the optimal tuning parameter (λ_min_ = 0.0392) was selected to identify the most significant features (Figure 5A). Second, in the RF analysis, the error rate stabilized when the number of decision trees reached 500 (Figure 5B). Based on the MeanDecreaseGini index, the top 10 features (all with importance scores >1.3) were selected as candidate markers (Figure 5C). Third, the Boruta algorithm was employed to distinguish all relevant features from random noise. The dynamic selection process and the final importance of confirmed attributes are illustrated in Figures 5D,E, respectively. Fourth, the GBM algorithm was utilized to rank the contribution of genes, identifying the top 10 key features (Figure 5F). Finally, SVM-RFE analysis was conducted to screen for the optimal gene subset that maximized classification accuracy (Figure 5G). To ensure the robustness of the biomarkers, we performed an intersection analysis of the candidate genes identified by these five algorithms. Ultimately, four overlapping hub genes (SRF, ATF3, ME1, and GADD45G) were identified as the core diagnostic signature for subsequent validation (Figure 5H).

### 3.5 Model construction and evaluation

**FIGURE 6:**
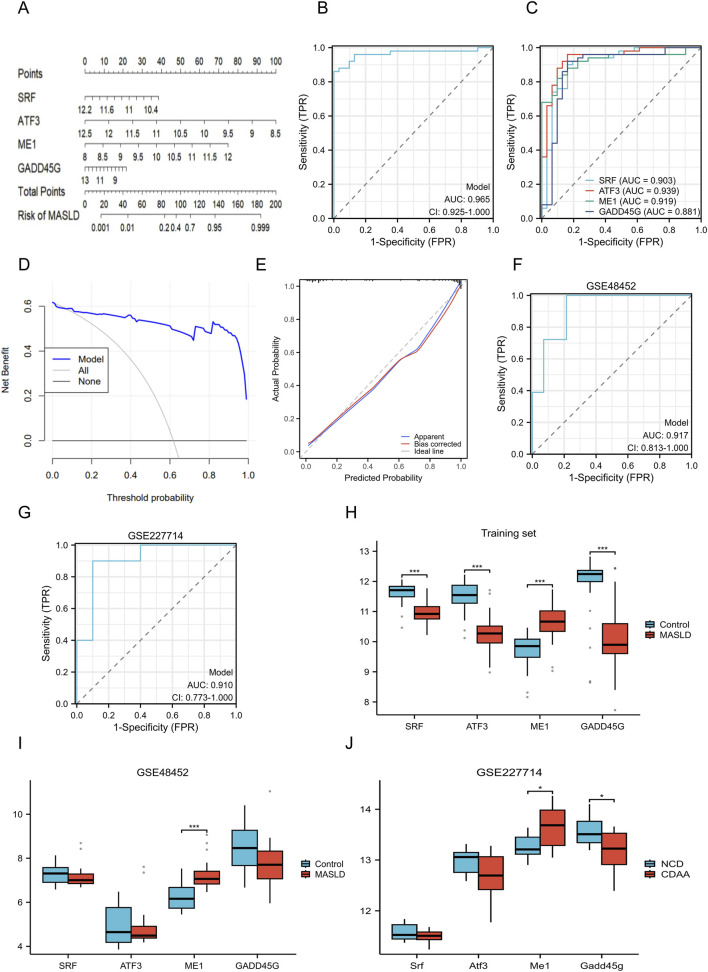
Construction and validation of the nomogram model for MASLD assessment. **(A)** Nomogram for estimating the probability of MASLD based on the four hub genes. The total points calculated from the four genes correspond to the likelihood of MASLD presence. **(B,C)** ROC curves evaluating the discrimination ability of the nomogram (AUC = 0.965) and individual genes (AUC >0.88) in the training set. **(D,E)** DCA and calibration plots assessing the clinical net benefit and prediction accuracy of the model. **(F,G)** External validation ROC curves in the human dataset GSE48452 (AUC = 0.917) and rat dataset GSE227714 (AUC = 0.910). **(H–J)** Differential expression levels of the four hub genes in the training set **(H)**, external validation dataset GSE48452 **(I)**, and GSE227714 **(J)**. *P < 0.05, ***P < 0.001. AUC, area under the curve; DCA, decision curve analysis; ROC, receiver operating characteristic; MASLD, metabolic dysfunction-associated steatotic liver disease.

Based on the four identified hub genes, we constructed a quantitative nomogram to estimate the risk probability of MASLD (Figure 6A). The model demonstrated excellent discrimination in the training set, achieving an AUC of 0.965 (95% CI: 0.925–1.000) (Figure 6B). Furthermore, the individual AUC values for all four genes exceeded 0.8, suggesting their robust capability as candidate biomarkers (Figure 6C). The calibration curve revealed high agreement between the predicted probability and actual observations (Figure 6E), indicating the model’s goodness-of-fit. Additionally, DCA curve demonstrated that the nomogram offered a higher net benefit compared to default strategies, suggesting its potential utility for MASLD assessment (Figure 6D). To further evaluate the robustness of these biomarkers, we performed validation in independent external datasets (human dataset GSE48452 and rat dataset GSE227714). The results were consistent with the training set, yielding AUCs of 0.917 and 0.910, respectively (Figures 6F,G). These findings reinforce the reliability of the four-gene signature across different species and cohorts, although further clinical verification is required. Finally, expression analysis confirmed consistent trends: SRF, ATF3, and GADD45G were significantly downregulated, whereas ME1 was upregulated in MASLD samples in the training set (Figure 6H), a pattern that was successfully corroborated in both external validation cohorts (Figures 6I,J).

### 3.6 GSEA and ssGSEA immune infiltration of the hub genes

**FIGURE 7:**
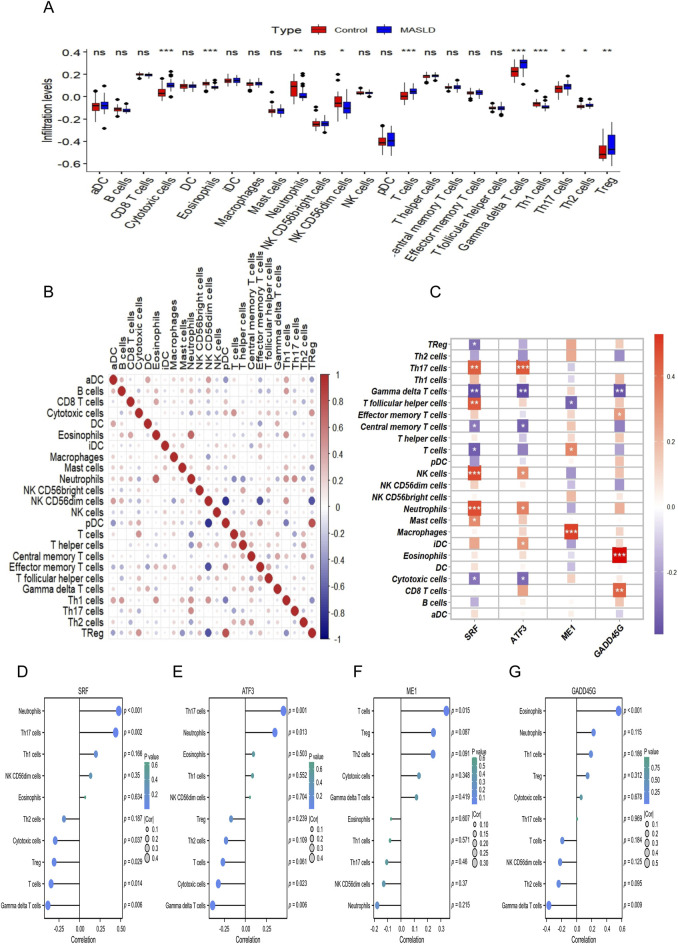
Evaluation of the immune microenvironment landscape and its association with the four hub genes. **(A)** Comparison of the infiltration levels of 24 immune cell types between the MASLD (blue box) and Control (red box) groups, calculated using the ssGSEA algorithm and analyzed by the *Wilcoxon rank-sum test*. **(B)** Correlation heatmap illustrating the interaction patterns among different immune cell types. Red indicates positive correlation, while blue indicates negative correlation. **(C)** Heatmap displaying the correlation analysis between the four hub genes and infiltrating immune cells. The color intensity reflects the strength of the correlation coefficient. **(D–G)** Lollipop charts detailing the correlations between the infiltration levels of specific immune cells and the expression of **(D)** *SRF*, **(E)** *ATF3*, **(F)** *ME1*, and **(G)** *GADD45G*. The size of the dots represents the Spearman correlation coefficient, and the color scale corresponds to the significance level. In the box plots, the central line represents the median, and the box limits indicate the IQR. *ns*, not significant, *P < 0.05, **P < 0.01, ***P < 0.001. MASLD, metabolic dysfunction-associated steatotic liver disease; IQR, interquartile range; ssGSEA, single-sample gene set enrichment analysis.

To comprehensively characterize the immune landscape of MASLD, we quantified the relative infiltration levels of 24 immune cell types using the ssGSEA algorithm. As shown in Figure 7A, the *Wilcoxon rank-sum test* revealed significant alterations in the immune microenvironment between MASLD and control samples. Specifically, ten immune cell populations exhibited significant differential abundance, including cytotoxic cells, eosinophils, neutrophils, NK CD56dim cells, T cells, γδT cells, Th1 cells, Th17 cells, Th2 cells, and Tregs (*P* < 0.05). To explore the potential link between the identified hub genes and immune dysregulation, we performed spearman correlation analysis. The correlation landscape between the four hub genes and the 24 immune cell types is visualized in Figure 7C. Notably, *SRF* showed the broadest immune engagement, significantly correlating with 10 distinct immune cell types. Detailed correlation profiles for specific cell types are presented in Figures 7D–G. We observed significant positive correlations between *SRF* and *ATF3* and proinflammatory subsets such as Th17 cells and neutrophils, suggesting a potential role in mediating hepatic inflammation. Additionally, *ME1* was positively correlated with T cells, while *GADD45G* showed a strong association with eosinophils. Furthermore, to elucidate the underlying biological mechanisms driven by these biomarkers, we conducted GSEA. The results indicated that the hub genes are critically involved in metabolic and inflammatory signaling pathways relevant to MASLD pathology. Specifically, *SRF* was significantly enriched in the “MAPK signaling pathway” as well as processes related to “glucose metabolism” and “triglyceride catabolism” (Supplementary Figure S1A). *ATF3* showed enrichment in the “insulin signaling pathway,” “cholesterol biosynthesis,” and “triglyceride biosynthesis” (Supplementary Figure S1B). *ME1* was primarily associated with the “PPAR signaling pathway” and lipid metabolism processes (Supplementary Figure S1C). Finally, *GADD45G* was enriched in “fatty acid metabolism,” “peroxisomal lipid metabolism,” and oxidative stress-related pathways such as “biological oxidation” (Supplementary Figure S1D). These findings collectively suggest that the four hub genes may modulate MASLD progression by orchestrating immune infiltration and regulating lipid metabolic networks.

### 3.7 *In vivo* and *in vitro* experimental validation

**FIGURE 8:**
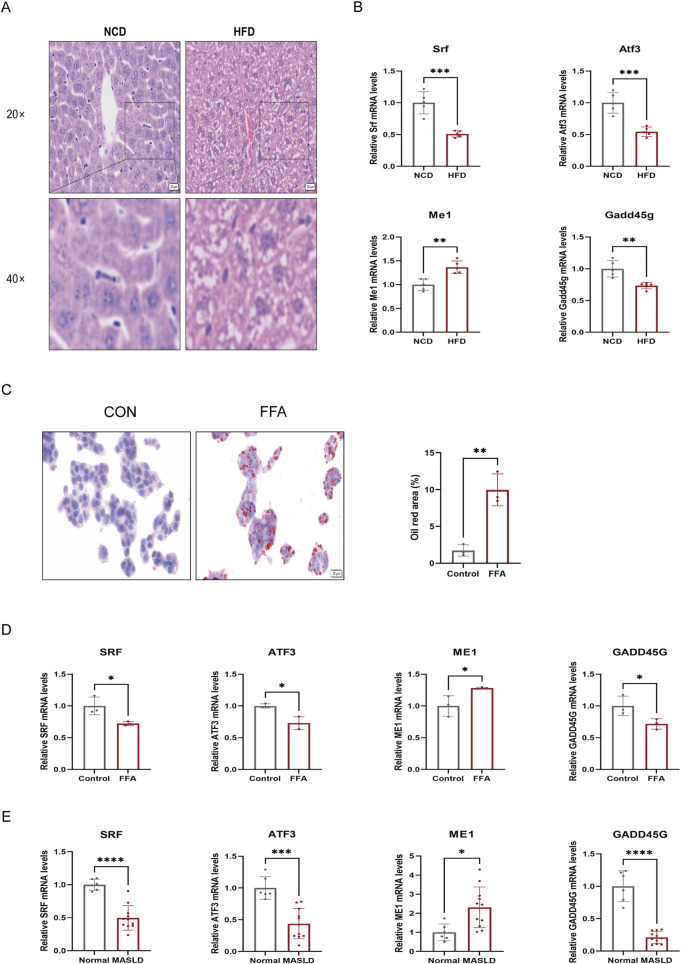
Experimental validation of the four hub genes in *vivo*, *in vitro*, and clinical samples. **(A)** Representative H&E staining images of liver tissues from NCD and HFD mice groups (×20 and ×40 magnification; *n* = 5 per group). **(B)** RT-qPCR analysis of *Srf, Atf3, Me1*, and *Gadd45g* mRNA expression levels in mouse liver tissues (*n* = 5 per group). **(C)** Representative Oil Red O staining images of HepG2 cells in the Control and FFA-treated groups. The bar chart shows the quantification of the Oil Red O positive area from three independent experiments (*n* = 3). **(D)** RT-qPCR analysis of the four hub genes in FFA-induced HepG2 cells compared to controls (*n* = 3 independent experiments). **(E)** Validation of the mRNA expression levels of *SRF, ATF3, ME1,* and *GADD45G* in clinical liver samples from Normal and MASLD patients. Data are presented as mean ± SEM. Statistical significance between two groups was determined using the unpaired Student’s t-test. *P < 0.05, **P < 0.01, ***P < 0.001, ****P < 0.0001. FFA, free fatty acid; HFD, high-fat diet; MASLD, metabolic dysfunction-associated steatotic liver disease; NCD, normal chow diet.

To validate the expression patterns of the four hub genes, we employed a comprehensive validation strategy encompassing animal models, cellular assays, and clinical liver samples. First, a MASLD mouse model was established using a high-fat diet (HFD). Quantitative assessment revealed that body weight, liver wet weight, and serum biochemical indices (ALT, AST, TC, and TG) were significantly elevated in the HFD group compared to the NCD group (Supplementary Figure S2C-H). Macroscopically, the livers of HFD-fed mice appeared enlarged and exhibited a pale yellow color indicative of steatosis (Supplementary Figure S2B). Histologically, H&E staining confirmed prominent macrovesicular steatosis and lipid accumulation in the hepatocytes of HFD-fed mice compared to the NCD group (Figure 8A). In accordance with our bioinformatic predictions, RT-qPCR analysis (*n* = 5 per group) demonstrated that the mRNA expression of *Srf, Atf3,* and *Gadd45g* was significantly downregulated in HFD-challenged livers, while *Me1* expression was markedly upregulated (Figure 8B).

Next, an *in vitro* model of hepatic steatosis was generated by treating HepG2 cells with FFA for 24 h. Oil Red O staining and subsequent quantitative analysis from three independent experiments revealed a significant increase in intracellular lipid droplets following FFA treatment (Figure 8C). Consistent with the *in vivo* findings, FFA stimulation led to a significant decrease in the mRNA levels of *SRF, ATF3*, and *GADD45G*, whereas *ME1* expression was significantly elevated (Figure 8D).

Finally, we corroborated these findings in clinical liver tissue samples from 11 patients with MASLD and six healthy controls (baseline characteristics are provided in Supplementary Table S3). Histological examination further verified severe macrovesicular steatosis in MASLD patients compared to healthy controls (Supplementary Figure S2A). The clinical data highly paralleled our experimental results: *ME1* expression was significantly higher in the MASLD group than in the healthy group, while *SRF, ATF3,* and *GADD45G* levels were significantly reduced (Figure 8E). Collectively, the consistent expression patterns observed across mouse models, cell lines, and human clinical samples reinforce the reliability of these four genes as robust biomarkers and suggest their pivotal roles in the metabolic dysregulation and progression of MASLD.

In summary, although all four hub genes showed varying degrees of dysregulation, *SRF* was ultimately prioritized for further investigation due to its strongest correlation with immune cell infiltration in our bioinformatic analysis and its most consistent downregulation across all experimental models. This robust association with the MASLD microenvironment and its stable expression pattern suggest that *SRF* may play a pivotal role in the disease’s progression. To deeply explore its biological significance, we integrated single-cell RNA sequencing and virtual knockout analysis to resolve its cell-type-specific distribution and predict functional consequences, which was further complemented by *in vitro* knockdown experiments to experimentally confirm the causal role of *SRF* in hepatocyte senescence and lipid accumulation.

### 3.8 Single-cell transcriptomic profiling reveals hepatocyte-specific downregulation of Srf and its predicted metabolic consequences in MASLD

**FIGURE 9:**
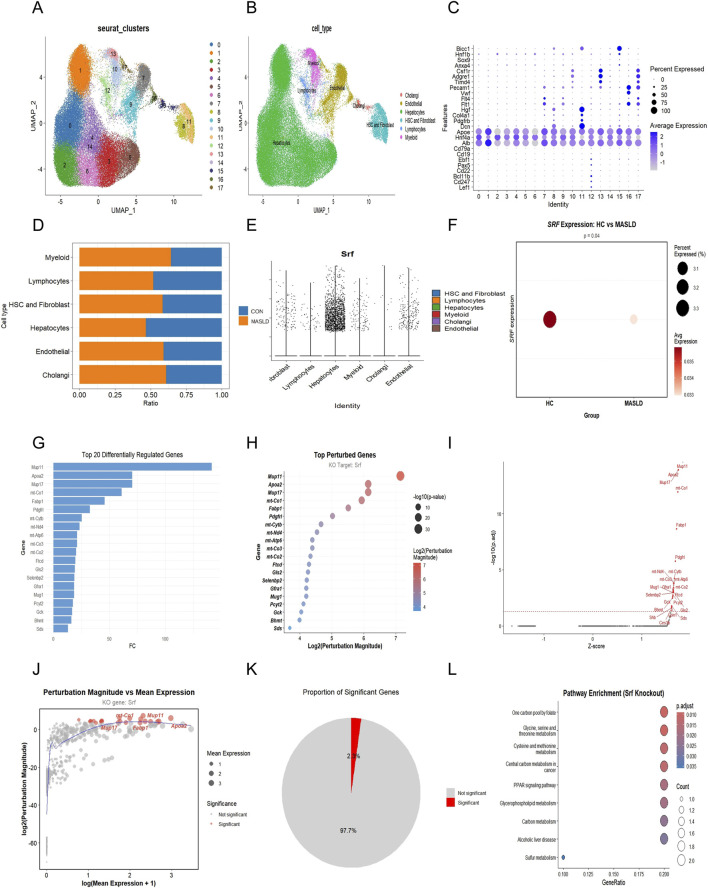
Single-cell expression profiling and functional perturbation analysis of Srf in MASLD. **(A)** UMAP clustering. Unsupervised clustering of intrahepatic cells, identifying 18 distinct cell clusters. **(B)** Cell type annotation. Identification of 6 major cell lineages, including Hepatocytes, Myeloid cells, and Lymphocytes, based on canonical markers. **(C)** Lineage-specific markers. Dot plot validating the expression of key marker genes across identified cell populations. **(D)** Cell proportion shifts. Comparison of relative cell type percentages between control and MASLD groups, showing hepatocyte loss and myeloid expansion. **(E)** *Srf* expression landscape. Violin plot demonstrating the predominant expression of *Srf* in the hepatocyte cluster. **(F)** Group-specific Srf expression. Comparative violin plot showing the significant downregulation of *Srf* in MASLD hepatocytes (*P* = 0.04). **(G)** Top regulated genes. Bar plot of the top 20 differentially regulated genes following virtual Srf knockout. **(H)** Top perturbed genes. Bubble plot illustrating the genes most sensitive to the loss of Srf function. **(I)** Volcano plot of perturbations. Distribution of upregulated and downregulated genes upon simulated Srf deficiency. **(J)** Manhattan-style plot of transcriptomic perturbations. Distribution of gene-level perturbation magnitudes across the hepatocyte expression spectrum upon *Srf* knockout. **(K)** Proportion of significant genes. Pie chart representing the fraction of the hepatocyte transcriptome significantly affected by *Srf* loss. **(L)** Pathway enrichment analysis. KEGG enrichment of *Srf*-regulated genes, highlighting the PPAR signaling pathway and metabolic dysfunction.

To characterize the cellular composition of the liver microenvironment, we performed our own independently generated single-cell RNA sequencing (scRNA-seq) on the established experimental models. Initially, metrics such as nCount_RNA, nFeature_RNA, and mitochondrial/hemoglobin content exhibited broad variation. To remove low-quality cells, we applied strict filtering criteria: cells with fewer than 500 or more than 4,000 detected genes, as well as those with >20% mitochondrial gene content, were excluded. This process significantly improved the distribution of QC metrics (Supplementary Figures S3A-S3D). Unsupervised clustering and UMAP visualization identified 18 distinct cell clusters (Figure 9A), which were subsequently annotated into six major cell lineages based on canonical marker genes: hepatocytes, myeloid cells, lymphocytes (T and B cells), HSC and fibroblasts, endothelial cells, and cholangiocytes (Figure 9B). The identity of each cell cluster was rigorously confirmed by the expression of lineage-specific markers shown in the dot plot (Figure 9C). Quantitative analysis of cell proportions revealed a dramatic remodeling of the liver landscape during MASLD progression. Compared to the control group, the MASLD group exhibited a significant contraction of the hepatocyte fraction, accompanied by a marked expansion of myeloid cells and HSC/fibroblasts (Figure 9D). This shift aligns with the pathological hallmarks of parenchymal loss, chronic inflammation, and fibrotic activation in MASLD. We then examined the expression of Srf across all identified cell types. The violin plot demonstrated that *Srf* is not uniformly distributed but is predominantly and robustly expressed in hepatocytes (Figure 9E). Notably, a comparison between groups showed that the expression level and the percentage of *Srf*-positive cells were significantly reduced in MASLD hepatocytes compared to the control group (Figure 9F, *P* = 0.04). This hepatocyte-specific downregulation suggests that Srf deficiency is a key event in the metabolic deterioration of the liver.

To further investigate whether this hepatocyte-specific downregulation of *Srf* is a passive consequence or a primary driver of metabolic dysfunction, we performed an *in silico* virtual knockout (vKO) analysis to simulate the transcriptomic impact of *Srf* loss. The perturbation identified the top differentially regulated downstream genes, including significant alterations in *Map1l*, *Apoa2*, and *Mup17* (Figures 9G–I). The distribution of perturbation magnitudes relative to mean expression confirmed that a substantial proportion of the hepatocyte transcriptome is sensitive to Srf depletion (Figures 9J,K). Our analysis revealed that 2.3% of the entire hepatocyte transcriptome exhibited significant sensitivity to *Srf* depletion (Figure 9K). The Manhattan-style perturbation plot (Figure 9J) and Pathway enrichment (Figure 9L) collectively suggest that Srf deficiency triggers a multi-dimensional stress response, characterized by mitochondrial dysfunction (e.g., *mt-Co1*), lipid toxicity (e.g., *Apoa2* and PPAR signaling), and epigenetic instability (One carbon metabolism). Given that these biological processes are well-recognized upstream triggers for the p53/p21-mediated senescence program, these high-resolution *in silico* findings strongly point toward Srf as a potential protective ‘molecular anchor’ against hepatocyte aging.

These computational predictions provided a robust mechanistic rationale for our subsequent experimental validations. While the *in silico* perturbations provided a high-resolution mechanistic rationale, the causal role of *SRF* in hepatocyte senescence and lipid accumulation required direct experimental confirmation. Therefore, we performed siRNA-mediated knockdown of *SRF* in a fatty acid-induced hepatocyte model to validate these computational predictions.

### 3.9 Downregulation of SRF exacerbates lipid deposition in hepatocytes

**FIGURE 10:**
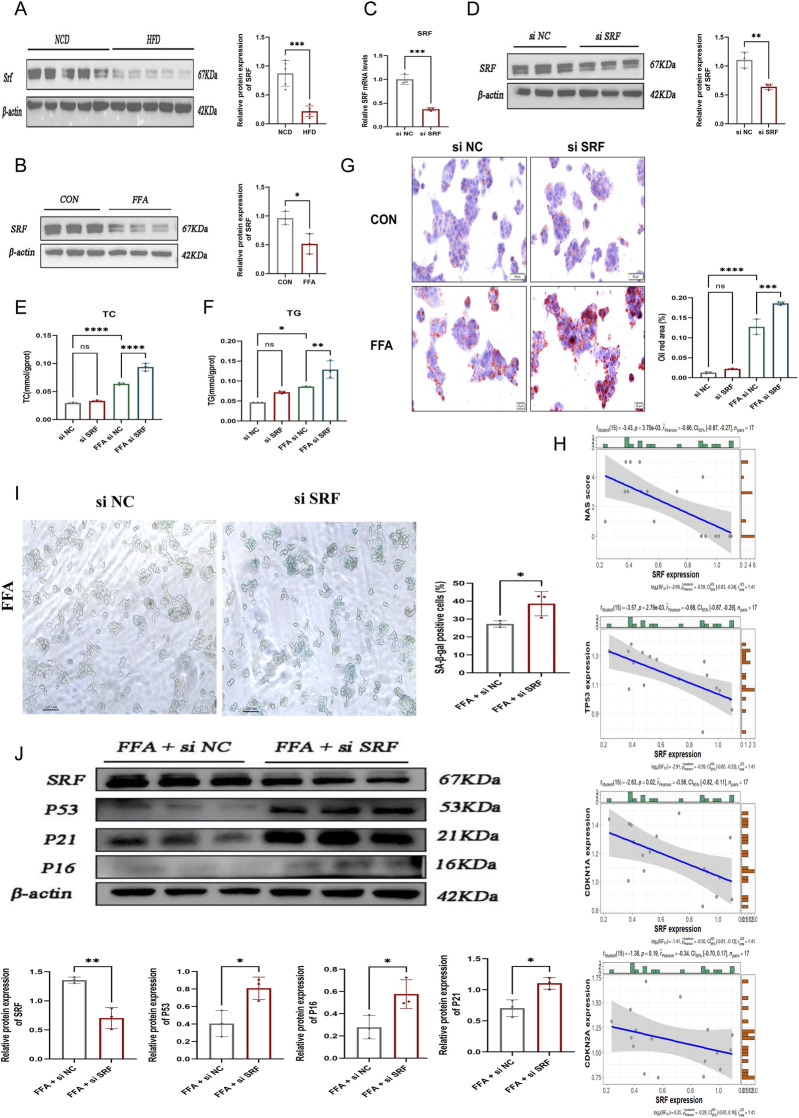
SRF deficiency drives lipid accumulation and hepatocyte senescence. **(A)** Western blot analysis and quantification of SRF protein expression in liver tissues from NCD and HFD mice. β-Actin was used as the loading control (*n* = 5 per group). **(B)** Western blot analysis of SRF protein levels in control and FFA-treated HepG2 cells (*n* = 3 independent experiments). **(C,D)** Verification of *SRF* knockdown efficiency by RT-qPCR **(C)** and Western blot **(D)** in HepG2 cells transfected with si-NC or *si-SRF*. Data were obtained from three independent experiments. **(E)** Representative Oil Red O staining images and quantitative analysis of lipid droplets in HepG2 cells. Cells were transfected with si-NC or *si-SRF* and treated with DMSO or FFA as indicated (*n* = 3 independent experiments). **(F,G)** Measurement of intracellular TC and TG contents in HepG2 cells under the indicated conditions (*n* = 3 independent experiments). **(H)** Correlation analysis between *SRF* expression and clinical parameters (NAS score, TP53, CDKN1A, and CDKN2A) in clinical liver samples (*n* = 17). **(I)** Representative images of SA-β-gal staining in FFA-induced HepG2 cells following transfection with si-NC or *si-SRF* (*n* = 3 independent experiments). **(J)** Western blot analysis and quantification of p53, p21, and p16 protein levels in HepG2 cells transfected with si-NC or si-SRF under FFA treatment (*n* = 3). Data are presented as mean ± SEM. Statistical significance was determined using the unpaired Student’s t-test (for two groups) or one-way ANOVA followed by Student–Newman–Keuls (for multiple comparisons). ns, not significant; *P < 0.05, **P < 0.01, ***P < 0.001, ****P < 0.0001. FFA, free fatty acid; HFD, high fat diet; NCD, normal chow diet; TC, total cholesterol; TG, triglyceride.

Given that *SRF* exhibited the most extensive correlation with immune cell infiltration among the identified hub genes, we prioritized it for in-depth functional validation to investigate its specific biological role in MASLD progression. First, we confirmed the protein expression of SRF in our established models. Western blot analysis showed that SRF protein levels were significantly decreased in both HFD-induced mouse livers (Figure 10A) and FFA-induced HepG2 cells (Figure 10B), consistent with our bioinformatic findings.

To functionally verify whether the loss of *SRF* contributes to MASLD progression, we performed loss-of-function assays using siRNA in HepG2 cells. RT-qPCR and Western blotting confirmed that the expression of *SRF* mRNA and protein was reduced by more than 50% in the si-SRF group compared to the si-NC group, verifying high knockdown efficiency (Figures 10C,D). Subsequently, we evaluated the impact of *SRF* silencing on lipid metabolism under FFA stimulation. Oil Red O staining revealed that *SRF* knockdown markedly aggravated intracellular lipid deposition compared to the si-NC group under FFA treatment (Figure 10E). Quantitative analysis further corroborated this finding, showing significantly elevated intracellular levels of TC and TG in *si-SRF* cells (Figures 10F,G).

Furthermore, we investigated the mechanistic link between *SRF* and senescence, we examined its relationship with clinical parameters in 17 MASLD patient samples. Correlation analysis (Figure 10H) revealed that *SRF* expression was negatively associated with the MASLD activity score (NAS) and showed a robust negative correlation with key senescence markers, including *TP53* (r = −0.68, *P* < 0.01) and *CDKN1A* (p21, r = −0.70, *P* < 0.01). Quantitative SA-β-gal staining showed a significant increase in the percentage of senescent cells following *SRF* knockdown under FFA stimulation (Figure 10I). Mechanistically, Western blot analysis demonstrated that *SRF* silencing led to a marked upregulation of p53 and p21 protein levels, whereas the change in p16 was less pronounced (Figure 10J). Collectively, these results suggest that *SRF* deficiency is a potential driver of MASLD pathology, which may aggravate hepatic steatosis by activating the p53/p21-dependent senescence program.

## 4 Discussion

MASLD has become one of the most common chronic liver diseases worldwide, and its incidence is closely associated with metabolic disorders such as obesity, metabolic syndrome, and type 2 diabetes mellitus (Wu et al., 2020). In recent years, the role of cellular senescence in the development and progression of MASLD has gradually attracted increasing attention (Baboota et al., 2022). Cellular senescence is a state of irreversible cell cycle arrest accompanied by the activation of the SASP, which releases proinflammatory and profibrotic factors, thereby exacerbating liver inflammation and fibrosis (Aravinthan et al., 2013).

In this study, we integrated four datasets from the GEO database, identifying 589 DEGs in the training set. By intersecting these with WGCNA modules and cellular senescence-related genes, we identified 46 CS-DEGs. Functional enrichment analysis revealed that these genes are involved in processes such as “fat cell differentiation” and the “TNF-α signaling pathway,” suggesting that cellular senescence may drive MASLD progression by regulating lipid metabolism and inflammatory responses. For example, activation of the TNF-α signaling pathway has been confirmed to promote lipid accumulation in hepatocytes and insulin resistance, while SASP factors (e.g., IL-6 and TGF-β) released by senescent cells may further exacerbate deterioration of the hepatic microenvironment (Mohallem and Aryal, 2021). Additionally, GO analysis highlighted that the enrichment of genes in the “endoplasmic reticulum lumen” and “transcription regulatory complex,” implying that endoplasmic reticulum stress (ERS) and transcriptional dysregulation may be key mechanisms linking senescence to MASLD pathology.

Unlike recent studies that rely on single screening methods or limit validation to expression levels (Luo et al., 2024; Wang et al., 2025), our study advances the field in two key aspects. First, we implemented a rigorous ensemble machine learning framework integrating five distinct algorithms (LASSO, RF, Boruta, GBM, and SVM-RFE) to minimize algorithm-specific bias and identify robust core targets. Second, we moved beyond correlation to causality by performing functional loss-of-function assays. Demonstrating that *SRF* knockdown directly exacerbates lipid accumulation and senescence establishes a specific causal link, distinguishing our findings from purely descriptive biomarker studies. Third, we employed single-cell RNA sequencing and *in silico* virtual knockout analysis to resolve the cell-type-specific distribution of *SRF*. This high-resolution approach allowed us to pinpoint hepatocytes as the primary site of *SRF* dysregulation and predict its functional impact at a granular level, providing a mechanistic bridge between bulk transcriptomic signatures and experimental phenotypes.

*SRF* (Serum Response Factor), located on chromosome 6p21.1, is a transcription factor that regulates cell growth, differentiation, and migration (Herbert et al., 2022). As a dimer, SRF binds to motifs containing the conserved CArG box [CC(A/T)6GG] and activates transcription (Miano, 2010). Previous studies have shown that *SRF* is downregulated in senescent hepatic stellate cells and that restoring *SRF* expression can alleviate fibrosis (Guo et al., 2023). The hepatocytes of MASLD patients usually exhibit a senescent state, with severe DNA damage and cell cycle arrest. Studies have shown that depletion of senescent cells in the body can reduce the degree of steatosis, while inducing hepatocyte senescence can promote hepatic lipid deposition (Ogrodnik et al., 2017). In our study, *SRF* was significantly downregulated in MASLD models and clinical samples. Aligning with previous reports that *SRF* deficiency exacerbates fibrosis in hepatic stellate cells, our results intriguingly showed that in hepatocytes, *SRF* knockdown promoted lipid accumulation and senescence. These combined findings suggest that *SRF* functions as a critical guardian of liver homeostasis, and its deficiency is a common pathogenic driver for both hepatic steatosis and fibrosis. Furthermore, we observed a strong positive correlation between *SRF* expression and NK cell infiltration (Figure 7C). Since NK cells are often functionally exhausted or reduced in MASLD patients, the downregulation of *SRF* may contribute to disease progression by fostering an immunosuppressive microenvironment, a novel mechanism that warrants further investigation (Sakamoto et al., 2021).

*ATF3* is a stress-inducible transcription factor involved in HDL and triglyceride metabolism, preventing atherosclerosis and MASLD progression (Gilchrist et al., 2006; Xu et al., 2021a; Xu et al., 2021b). *ME1* catalyzes the conversion of malate to pyruvate, participating in fatty acid synthesis and redox balance (Zhu et al., 2020; Al-Dwairi et al., 2012). *GADD45G* is involved in DNA damage response; its low expression may impair the hepatocyte’s ability to repair DNA damage, accumulating cellular stress linked to senescence (Zeng et al., 2023).

Our bioinformatic analysis *via* ssGSEA provided a preliminary overview of the immune landscape in MASLD, suggesting potential shifts in the infiltration of several immune subsets. We observed that certain cell types, including γδ T cells, Th17 cells, and Tregs, showed altered infiltration patterns associated with the disease state. Notably, *SRF* expression exhibited a significant correlation with the inferred abundance of γδ T cells. While γδ T cells represent a small fraction of the hepatic immune pool, they have been implicated in modulating liver inflammation in various metabolic models (Zhao et al., 2018; Li et al., 2022; Han et al., 2023; Torres-Hernandez et al., 2020). In this context, the observed correlation between *SRF* downregulation and immune cell dynamics, such as the predicted exhaustion of neutrophils and NK CD56dim cells, hints at a potential link between *SRF* loss and a dysfunctional immune microenvironment (Zhao et al., 2020; van der Windt et al., 2018). However, as these immune signatures were derived from computational deconvolution of bulk transcriptomic data, these associations should be interpreted as hypothesis-generating. Future experimental studies, such as flow cytometry or spatial transcriptomics, are required to definitively characterize the interplay between *SRF* and the MASLD immune milieu.

Despite the comprehensive integration of bioinformatics analysis and experimental validation, several limitations must be acknowledged. First, the sample sizes of both our clinical liver tissues and experimental animal cohorts were relatively modest, which may limit the statistical power of validating the diagnostic value of the four hub genes. The clinical diagnostic potential of *SRF* and its identified hub genes requires further validation in larger-scale, prospective clinical trials. Future research incorporating diverse ethnic populations, varied clinical stages, and longitudinal follow-up data will be essential to rigorously establish the sensitivity and specificity of *SRF* as a robust, non-invasive biomarker for the early diagnosis and prognostic monitoring of MASLD. Second, while *SRF* knockdown confirmed a clear phenotype, the precise molecular mechanisms remain to be further mapped. To bridge this gap, we integrated single-cell RNA sequencing with *in silico* virtual knockout analysis to predict the transcriptomic alterations following *SRF* loss, providing a high-resolution mechanistic framework. Nevertheless, the specific upstream regulators and downstream signaling axes linking *SRF* to lipid metabolism and senescence warrant further experimental validation in future studies. Third, the immune infiltration landscape was estimated using bioinformatics algorithms (ssGSEA) based on bulk transcriptomic data. The lack of validation *via* orthogonal methods (e.g., flow cytometry or immunohistochemistry) implies that these results should be interpreted as predicted patterns rather than confirmed functional phenotypes. These associations require further verification in future experimental studies.

## 5 Conclusion

In conclusion, our study identifies *SRF* as a potential candidate biomarker for senescence in MASLD-associated senescence. While our functional assays and multi-platform validations provide strong preliminary evidence, further large-scale clinical trials are required to establish its definitive utility in routine clinical diagnostics.

## Data Availability

The datasets presented in this study can be found in online repositories. The names of the repository/repositories and accession number(s) can be found in the article/Supplementary Material.
